# Distribution and evolution of stable single α-helices (SAH domains) in myosin motor proteins

**DOI:** 10.1371/journal.pone.0174639

**Published:** 2017-04-03

**Authors:** Dominic Simm, Klas Hatje, Martin Kollmar

**Affiliations:** 1 Group Systems Biology of Motor Proteins, Department of NMR-based Structural Biology, Max-Planck-Institute for Biophysical Chemistry, Göttingen, Germany; 2 Theoretical Computer Science and Algorithmic Methods, Institute of Computer Science, Georg-August-University Göttingen, Göttingen, Germany; Consejo Superior de Investigaciones Cientificas, SPAIN

## Abstract

Stable single-alpha helices (SAHs) are versatile structural elements in many prokaryotic and eukaryotic proteins acting as semi-flexible linkers and constant force springs. This way SAH-domains function as part of the lever of many different myosins. Canonical myosin levers consist of one or several IQ-motifs to which light chains such as calmodulin bind. SAH-domains provide flexibility in length and stiffness to the myosin levers, and may be particularly suited for myosins working in crowded cellular environments. Although the function of the SAH-domains in human class-6 and class-10 myosins has well been characterised, the distribution of the SAH-domain in all myosin subfamilies and across the eukaryotic tree of life remained elusive. Here, we analysed the largest available myosin sequence dataset consisting of 7919 manually annotated myosin sequences from 938 species representing all major eukaryotic branches using the SAH-prediction algorithm of Waggawagga, a recently developed tool for the identification of SAH-domains. With this approach we identified SAH-domains in more than one third of the supposed 79 myosin subfamilies. Depending on the myosin class, the presence of SAH-domains can range from a few to almost all class members indicating complex patterns of independent and taxon-specific SAH-domain gain and loss.

## Introduction

Helices, which are not buried within globular structures or coiled-coil helical dimers, usually need networks of charge interactions for stabilization in water [1–5]. In the late 1980^th^ and early 1990^th^ many studies have been performed using poly-alanine peptide models aiming to resolve the conditions for helix formation and stabilization. Different amino acids (mainly aspartic acid, glutamic acid, lysine and arginine, but in some cases also glutamine) were introduced into these peptides alone and in all possible combinations at varying distances. The corresponding peptides were synthesised, their α-helicity experimentally determined by, for example, circular dichroism, and stabilization energies were obtained by fitting models to the data. Although poly-alanine peptides adopt α-helical conformations when sparsely interrupted by lysine [6], arginine [7] and glutamine residues [8], helices are especially stabilized by charged interactions (salt bridges) between residues at (*i*, *i+3*) and (*i*, *i+4*) spacing [1–3] and hydrogen-bonding interactions between polar/charged residues at (*i*, *i+3*) and (*i*, *i+4*) [4,5]. Additional stability is obtained through networks of oppositely charged residues in (*i*, *i+3*, *i+6*), (*i*, *i+3*, *i+7*), (*i*, *i+4*, *i+7*), or (*i*, *i+4*, *i+8*) distances [9,10]. In addition to these poly-alanine based peptides, studies have been performed on peptides with complex amino acid distributions [11,12]. However, each study used different combinations of residues and non-physiological experimental conditions were applied (e.g. salt concentrations).

Studies on natural proteins known to contain stable single α-helices (SAHs) have shown that the respective sequence regions mainly consist of repeated patterns of negatively and positively charged residues [13–18]. In congruence with the results of the poly-alanine based analyses, repeats of four negatively and four positively charged residues form the most stable α-helices, while peptides with repeats of two residues do not show helical content [15,19]. Genome-wide searches for SAH-domains revealed their presence in bacteria, archaea, and eukaryotes, with the largest number of potential SAH-domains identified in mammals [17,20]. Predictions of SAH-domains in human proteins range from 0.25% to 0.4% [20,21], of which many belong to the cytoskeletal and motor proteins.

Myosins are a diverse protein family characterised by a large motor domain, which in most subfamilies contains an ATP-hydrolysis and an actin-binding site, and extended so-called tail domains located both N- and C-terminal to the motor domain [22]. Many myosins contain regions with one or multiple IQ-motifs C-terminal to the motor domain for binding calmodulin-family proteins that together act as a lever to transmit the power of the working stroke into displacement of the tail alongside the actin-filaments. Mammalian myosins from the class-6 and class-10 subfamilies have experimentally been shown to contain SAH-domains subsequent to the IQ-motif regions functioning as extended levers and constant force springs [14,17,23,24]. However, myosin tail architectures are very divergent even within subfamilies and a comprehensive analysis of SAH-domains in myosins based on deep taxonomic sampling across the supposed 79 subfamilies is still missing. Here, we analysed the largest available myosin dataset and identified putative SAH-domains in more than one third of all subfamilies. Depending on the myosin class, the presence of SAH-domains can range from a few to almost all class members indicating complex patterns of independent and taxon-specific SAH-domain gain and loss.

## Results and discussion

### Composition of the SAH-score

Because of the different combinations of residues and experimental conditions used in studies determining peptide stabilization energies, it is difficult to obtain and tabulate experimentally validated stabilization energies for all possible combinations of amino acids in all positions. We tried to develop a score reflecting the stability of a single α-helix compared to random coils and helices stabilized by interactions with other structural elements (e.g. within a protein structure or by binding to another protein via a coiled-coil motif). To this end, published stabilization energies [1–5,10] were compiled and set into relation to define stabilization values for all types of salt bridges and hydrogen-bonding interactions. We distinguish three types of stabilizations, weak, medium and strong, and classified all possible amino acid interactions accordingly (S1 Fig). In addition to binary interactions, an additional stabilization value is added for networks of at least three residues of oppositely charged residues in (*i*, *i+3*, *i+6*), (*i*, *i+3*, *i+7*), (*i*, *i+4*, *i+7*), or (*i*, *i+4*, *i+8*) distances. In contrast, networks of hydrophobic residues at distances (*i*, *i+3*, *i+7*) would provide a hydrophobic seam around the helix and might cause dimerisation by coiled-coil formation. Such hydrophobic networks are thought to be destabilizing and are accounted for by negative stabilization values. In addition to these values describing interactions, we assign alanines positive stabilization and glycines and prolines negative stabilization values, according to their helix promoting and helix braking characteristics, respectively. All stabilization values within a given sequence window are summed up and the sum is subsequently normalized against an idealised SAH-domain containing only strong interactions to compute an SAH-score for the central amino acid of the sequence window [25].

For comparison, we computed SAH-scores for four different window sizes, specifically 14, 21, 28, and 49 (about half a pitch of a two-stranded coiled-coil [26,27]). Small window sizes allow detection of relatively short SAH-domains (down to roughly four helix turns) but in these cases single or few residues can have large effects on the SAH-score both in terms of detecting false positives (coiled-coil regions often contain stretches with high percentages of charged residues) and missing true positives (one or few hydrophobic residues are well accommodated in stable single α-helices although they do not contribute to the SAH-score as defined above). With the largest window (49 amino acids, around 14 helical turns) the effect of single or few residues on the SAH-score is diminished but short SAH-domains might not be detected. These SAH-scores considerably fluctuate from residue to residue. Independent of score fluctuations, SAH-domains as structural entities are uninterrupted helices. However, similar to coiled-coil regions SAH-domains do not have distinct borders such as globular protein domains and might have any length. Therefore, specifying SAH-domain start and end positions as well as the transition point from non-SAH-domain to SAH-domain are a matter of definition.

### Determining SAH-domains in myosins

We obtained 7919 manually annotated myosin sequences from 938 species from CyMoBase (Kollmar and Mühlhausen, submitted), of which 7675 sequences contain tail regions with varying degree of completeness (6744 tails are complete, in 531 tails short sequence regions are missing, 490 tails are fragmented). The minimum length of a SAH-domain was set to 14 amino acids (around four helical turns). Every residue within a SAH-domain should have a SAH-score above a given threshold (here: 0.25). Known coiled-coil regions from muscle myosin heavy chain proteins, tropomyosins, and keratins clearly have lower SAH-scores, while known SAH-domains such as those from class-6 and class-10 myosins, GCP60, M4K4, INCENP, and Caldesmon-1 [13,14,16,18] have considerably higher SAH-scores. Therefore, a threshold of 0.25 for the SAH-score seemed reasonable. We allowed 20 percent of the scores of a putative SAH-domain to be below the threshold to avoid multiple successive regions interrupted by just one or two residues. Such residues within SAH-domains with SAH-scores below the threshold are, however, rare. Only 247 (1.2%) of 20449 [‘high’ SAH-domain-score cut-off] / 580 (1.7%) of 35002 [‘low’ SAH-domain-score cut-off] residues (numbers based on computing SAH-scores with a 14 amino acid window) within proposed SAH-domains have SAH-scores below the threshold.

**Fig 1:**
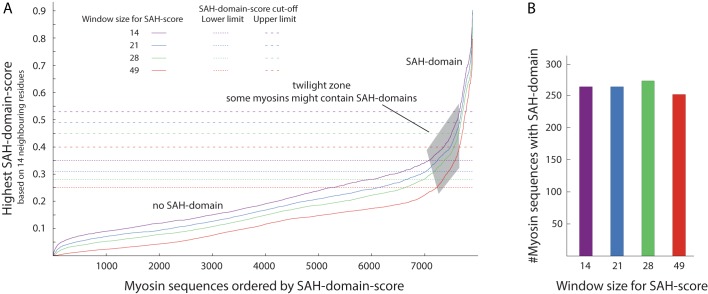
SAH-domains in myosins. A) For each myosin sequence, we determined the region with the highest SAH-domain-score, which is the average of the SAH-scores of 14 neighbouring amino acids. For comparison, we used SAH-scores computed for four different amino acid window sizes for SAH-domain-score determination. SAH-domain-score cut-offs were derived by sorting all sequences by SAH-domain-scores and determining the largest change in SAH-domain-score differences between two neighbouring sequences using kernel-regression (S2 Fig). B) Number of putative SAH-domains with minimum length of 14 amino acids in dependence of amino acid window sizes used to determine SAH-scores.

To obtain comparable scores for entire SAH-domains but to avoid bias by peak values, we define the *SAH-domain-score* as the highest average of the SAH-scores of any 14 neighbouring amino acids within each SAH-domain. The SAH-domain-score is dependent on the size of the sequence window for computing each amino acid’s SAH-score (Fig 1A, S1 Table). The distribution of the SAH-domain-scores does not indicate a clear distinction between SAH-domains and non-SAH-regions. This is the result of the low sequence and structural complexity of stable single α-helices, which allows all types of possible combinations of SAH-score-contributing amino acids at distances allowing charged and polar interactions. However, there is a clear region indicating unambiguous SAH-domains, and a large area indicating the absence of SAH-domains (Fig 1A). Sequences in the twilight zone might represent regions with high content of charged residues but nevertheless buried in tertiary and quaternary structures, or short single α-helices with atypically low numbers of charged and polar residues. It has been demonstrated that single amino acid mutations can cause small domains to switch between α-helix and β-sheet structures [28,29], and also cause coiled-coil segments to switch between different oligomeric states [30,31]. Accordingly, experimental evidence is needed to finally reveal the *in vivo* oligomeric state of the putative SAH-domains in the twilight zone. For further analyses, we use those SAH-domain-scores as cut-off, at which the changes in the increase of the score are highest (S2 Fig; ‘high’ SAH-domain-score cut-off). Applying these cut-offs, about 260 SAH-domains are detected for all window sizes of 14, 21, 28 and 49 amino acids (Fig 1B). The beginning of the twilight zone is defined by the first major change in the increase of the score (S2 Fig; ‘low’ SAH-domain-score cut-off).

### Distribution of SAH-domains across myosin classes

**Fig 2:**
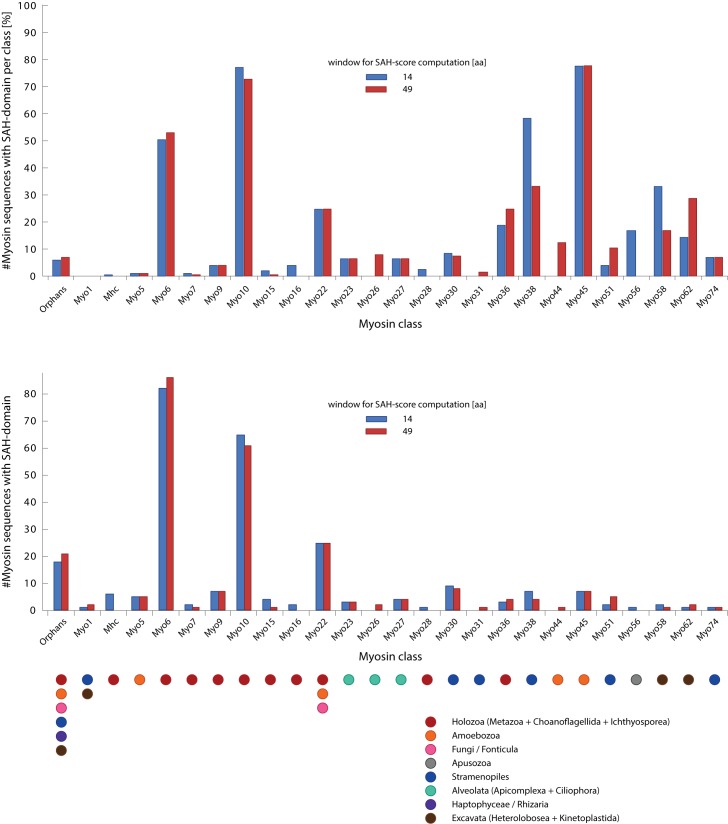
Distribution of SAH-domains with respect to myosin classes. The analysis of the percentage of myosins with SAH-domain per myosin class showed few classes, in which most or all myosins contain SAH-domains, while in most classes SAH-domains are present in only few, taxonomically restricted myosins. For comparison, the total number of myosins with SAH-domain is shown for each class. The taxonomic distribution of the myosins with SAH-domains is indicated at the bottom for each class. Note, that the taxa represent the first occurrence of respective myosins with SAH-domain, which is not always identical to the first occurrence of the respective myosin class. Subsequently, the SAH-domains were independently lost in many subtaxa so that the respective myosin motor domain and SAH-domain combination is not present in every extant species. For comparison, the distribution of SAH-domains with respect to myosin classes using the low SAH-domain-score cut-off is shown in S3 Fig.

**Fig 3:**
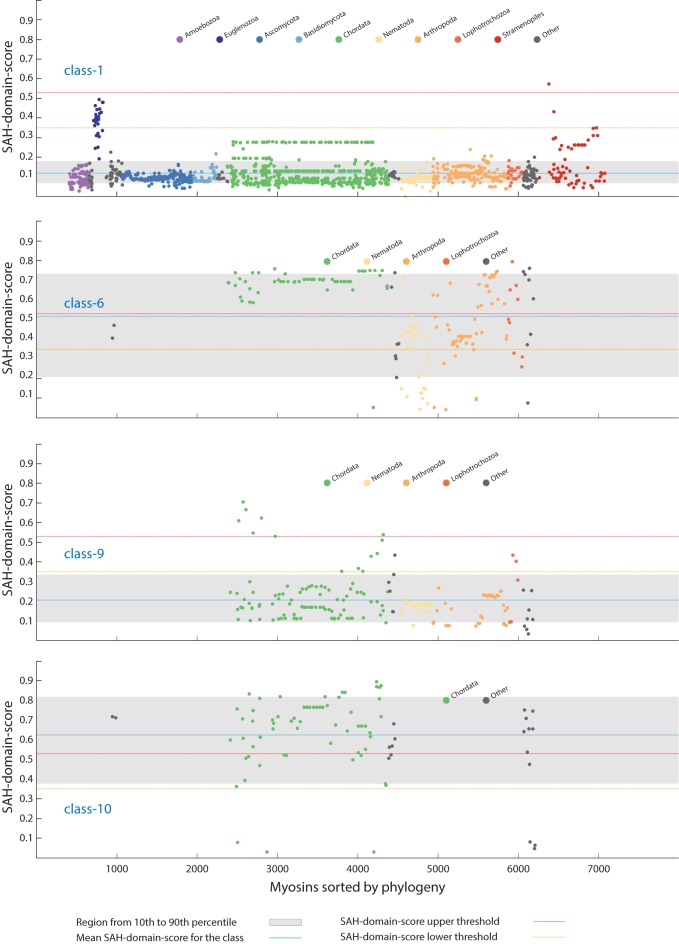
Phylogenetic distribution of the myosins. All myosins were sorted by taxonomy, and the highest SAH-domain-score for each myosin was plotted per class. Here, four examples based on the 14 amino acid window size for computing the SAH-score are shown representing classes with independent SAH-domain gain in late-branching taxa (class-1 and class-9), a class with multiple independent SAH-domain loss (class-6), and a class with all myosins containing SAH-domains (class-10; the sequences with SAH-domain-scores below 0.1 miss the putative SAH-domain regions because of genome and transcriptome assembly gaps). Major taxa are indicated by colour for better orientation. The phylogenetic distribution of all SAH-domains sorted by classes is shown in S4 and S5 Figs (window sizes of 14 and 49 amino acids, respectively, for computing the SAH-score).

SAH-domains were identified in 265 myosins (window size of 14 amino acids) from 25 myosin classes (32% of all classes). We also identified SAH-domains in 18 orphan myosins (Fig 2). The evaluation of the percentage of myosins with SAH-domain per class shows a tripartite distribution: A) A group of four classes, where most members contain SAH-domains, B) a group of about five classes with 15–40% of the myosins containing SAH-domains, and C) the remaining classes, of which only a small percentage or none of the class members contain SAH-domains (Fig 2). The classes with predominantly myosins with SAH-domains comprise the holozoan class-6, the opisthokont class-10, the stramenopiles class-38, and the amoebozoan class-45. The class-10 and the class-45 myosins are the only myosins where all members have SAH-domains (some class-10 SAH-domains have scores shortly below the high SAH-domain-score cut-off, and for some the respective regions comprising the SAH-domains are missing in the genome assemblies, Fig 3). Comparison of the SAH-domain predictions based on the 14 and 49 amino acid window sizes shows that most of the SAH-domains are long and detected with both window sizes (e.g. those of the class-6, class-10 and class-45 myosins), while in some classes such as the class-16, class-38, class-56, and class-58 the SAH-domains are particularly short (Fig 2 and S3 Fig). In contrast to the results obtained with other algorithms [20], SAH-domains in homologous myosins of closely related species (e.g. all mammals) were consistently detected.

### Gain and loss of SAH-domains in myosin classes

The current myosin data indicate that the last eukaryotic common ancestor only contained a class-1 myosin, of which members are present in almost all extant eukaryotes (the major exceptions are the plants and alveolates), and a second myosin of unknown class, which most probably had been the ancestor of all other classes [22]. The phylogenetic distribution of the classes with myosins containing SAH-domains implicates that the SAH-domains have been added to the tail domain architectures independently of each other. Within class-1 myosins, SAH-domains were detected in members of the Excavata and Stramenopiles taxa, also indicating independent gain. In the other, taxon-restricted classes, both independent gain in late-diverging branches and secondary loss were observed. Examples for independent gain are the SAH-domains in frog and fish Myo9B subtypes and the acorn worm *Saccoglossus kowalevskii* Myo9, examples for secondary SAH-domain loss events are the absence of SAH-domains in Platyhelminthes and many nematode class-6 myosins (Fig 3 and S4 and S5 Figs).

### Characteristics of SAH-domains in myosins

**Fig 4:**
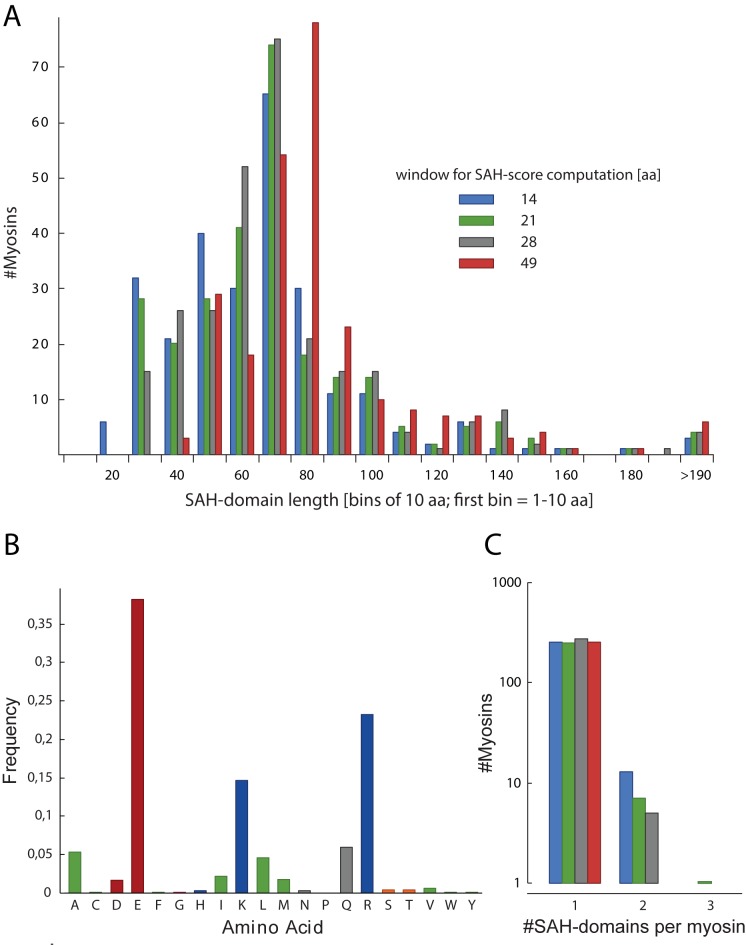
Characteristics of SAH-domains in myosins. A) Analysis of the length of SAH-domains. For better presentation, all SAH-domains with lengths within bins of ten amino acids were summed. B) Amino acid distribution counted over all 265 SAH-domains determined with the 14 amino acid window size SAH-score. C) Analysis of the number of SAH-domains per myosin.

The SAH-domains are on average 37–54 (low SAH-domain-score cut-off) / 62–81 (high SAH-domain-score cut-off) amino acids long, depending on the window size for SAH-score computation (Fig 4A). There is no preference for a certain SAH-domain length, and the number of myosins with SAH-domain lengths up to 80 amino acids is relatively constant. However, SAH-domains longer than 80 amino acids are rare. The longest SAH-domains of about 200 residues have been identified in cryptosporidian class-26 myosins. The lengths of the corresponding uninterrupted regular α-helices would be about 300 Å. Thus, the myosin SAH-domains have on average two-third of the mean-length of the average SAH-domains of all proteins, which are supposed to be about 75 residues long (according to protein sequences available in SwissProt and UniProt in 2012 [20]), although the distribution of number of sequences versus SAH-domain lengths is very similar [20].

The proposed SAH-domains contain only amino acids with high helical propensity [32], supporting that we identified only regions forming uninterrupted α-helices (Fig 4B). About 76% of the residues comprise charged amino acids with equal numbers of residues with positive and negative character, which is similar to the distribution of charged residues in a set of 47 proteins with supposed SAH-domains derived from SwissProt [16]. The percentage of charged residues is more than twice as high as observed in coiled-coil domains [21]. Compared to the amino acid distribution in α-helices in general [32] and coiled-coil regions in particular [16], hydrophobic residues are highly under-represented (only about 9.6% in the detected SAH-domains). These findings strongly support that the predicted SAH-domains are not able to oligomerize but instead comprise real stable single α-helices. A few hydrophobic residues might well be accommodated within SAH-domains through hydrophobic interactions with the hydrophobic parts of the arginine and lysine side chains [33].

### Functional implications

The importance of the SAH-domains as functional modules providing stiffness and modulating length of the lever in myosins has already been discussed in detail [16,20,21,23,34–36]. Our data show that the presence of SAH-domains in myosins is not myosin class-dependent but the result of a complex history of taxon- and species-specific gain and loss events. The only exceptions are the class-10 and class-45 myosins, of which all homologs identified to date contain SAH-domains. In all other cases, the myosins of interest have to be inspected manually. The data available in S1 Table and presented in S4 and S5 Figs could help in the evaluation. The SAH-domain-score cut-offs used throughout this study are rather conservative. Thus, although there might be few exceptions the SAH-domains with scores above these cut-offs can be regarded as veritable SAH-domains. This does not inevitably mean that the respective myosins are monomers. Dimerisation might happen through additional coiled-coil regions or other dimer-promoting domains. At least, coiled-coil predictions overlapping the SAH-domains as presented in the S1 Table can be regarded as mis-predictions caused by the intrinsic problems of all available coiled-coil prediction software to distinguish coiled-coils from SAH-domains [25].

### Examples for SAH-domains and twilight cases

**Fig 5:**
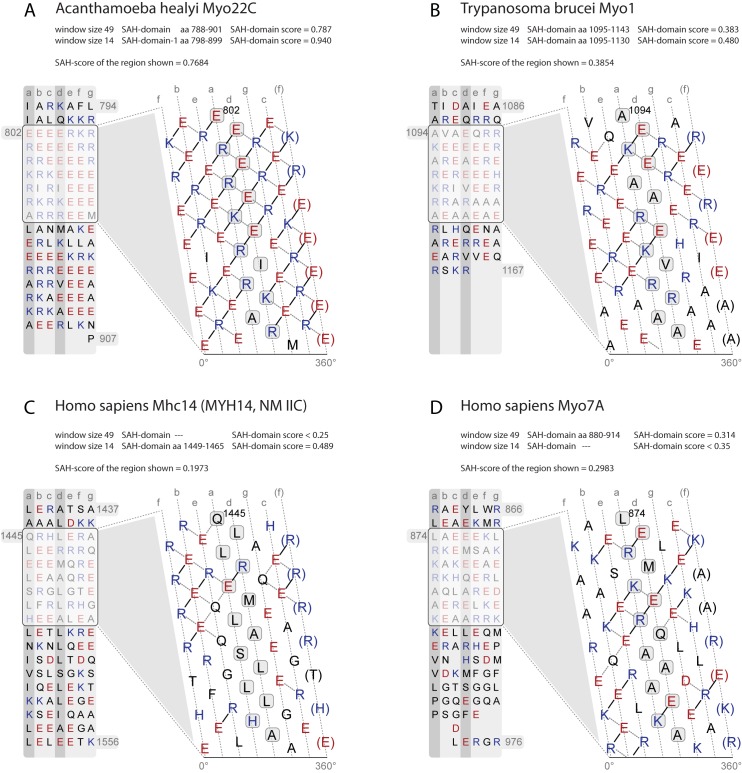
Examples of SAH-domains, and regions probably not containing SAH-domains. A) SAH-domain of *Acanthamoeba healyi* Myo22C, which is among the SAH-domains with the highest SAH-domain-scores. B) Example for a short SAH-domain, which is only detected with the 14, 21, and 28 amino acid window sizes for computing SAH-scores. This short SAH-domain is located C-terminal to the class-1 myosin-specific MyTH1 domain and therefore not part of the lever. Its putative function is to spatially separate the MyTH1 domain from another small domain or protein interaction motif of unknown function at the C-terminus. C) Example for a short SAH-domain with a SAH-domain-score in the twilight zone. This highly charged region is unique to mammalian Mhc14 class-2 myosins (MYH14, non-muscle myosin 2C; S6 Fig) and interrupts the long coiled-coil filament-forming region of the muscle myosins. The subsequent C-terminal sequence contains a large number of amino acids with high helix-breaking propensity such as glycines and serines. The putative function of the short SAH-domain and the following glycine-rich region might be to open up the coiled-coil. D) Example of a region rich in charged amino acids but with an SAH-domain-score in the range of non-SAH-domains.

In the following, we present examples for veritable SAH-domains and twilight-zone cases (Fig 5). The Acanthamoeba class-22 myosins contain the most ideal SAH-domains with dense networks of potential charged interactions only sporadically interrupted by small (e.g. alanines) and hydrophobic (e.g. isoleucines and leucines) residues (Fig 5A). A few hydrophobic residues can well be accommodated in SAH-domains through hydrophobic interaction with the hydrophobic parts of lysine and arginine residues [33].

The Kinetoplastid class-1 myosins contain unique tail architectures consisting of, from N- to C-terminus, an IQ motif, a WW, a MyTH1, a FYVE, and a subsequent short SAH-domain. This short SAH-domain is around 28 residues long (Fig 5B) and its function is most probably the spatial separation of the FYVE domain from a short C-terminal domain or protein interaction motif.

A short region in the tails of the mammalian Mhc14 class-2 myosins (MYH14, non-muscle myosin 2C) represents a twilight case (Fig 5C). This region is not present in other vertebrate Mhc14 orthologs and has evolved in the ancient mammalian Mhc14 (S6 Fig). The SAH-domain-score of this region is slightly below our conservative cut-off. Such a putative short SAH-domain within an extended coiled-coil domain is supposed to have a different function then causing monomerization. Rather, its function could be to open up and interrupt the coiled-coil region allowing local unwinding or bending of the α-helices similar to the unstable coiled-coil region of the N-terminal S2 (subfragment-2) domains of the skeletal muscle myosins [37]. This hypothesis is supported by the presence of many residues with strong helix-breaking propensity such as glycines and serines directly C-terminal to the putative SAH-domain (S6 Fig). The corresponding regions in the other mammalian muscle myosin homologs contain a conserved tryptophan at a “d” position (S6 Fig). This is one of the two conserved tryptophans in the muscle myosin rod region that were shown to be appreciably exposed to solvent and to be located in the least stable parts of the fibrous region [38–40]. Introducing a highly charged region in mammalian Mhc14 myosins could be an alternative solution to destabilize this part of the coiled-coil region.

The monomeric nature of the *Drosophila* Myo7A myosin *in vitro* [41,42] and the accumulation of charged residues in the region subsequent to the IQ motifs suggested this region also representing a SAH-domain [36]. However, we only observed veritable SAH-domains in cnidarian and placozoan class-7 myosins, and the short putative SAH-domains in *Drosophila* Myo7B myosins represent twilight cases (S1 Table, S4 and S5 Figs). The highest SAH-scores determined in all other class-7 myosins including *Drosophila* and human Myo7A are far below the upper SAH-domain-score cut-offs. The regions C-terminal to the IQ-motif region in class-7 myosins clearly do not contain hydrophobic residues ordered in a heptad pattern needed for coiled-coil formation (Fig 5D). However, these regions also do not have the extended and dense networks of charged amino acids characteristic of SAH-domains. These regions might therefore represent divergent types of SAH-domains not described and detected with current methods, or be part of a larger domain such as a helical bundle.

### Myosins with unusual lever architectures

**Fig 6:**
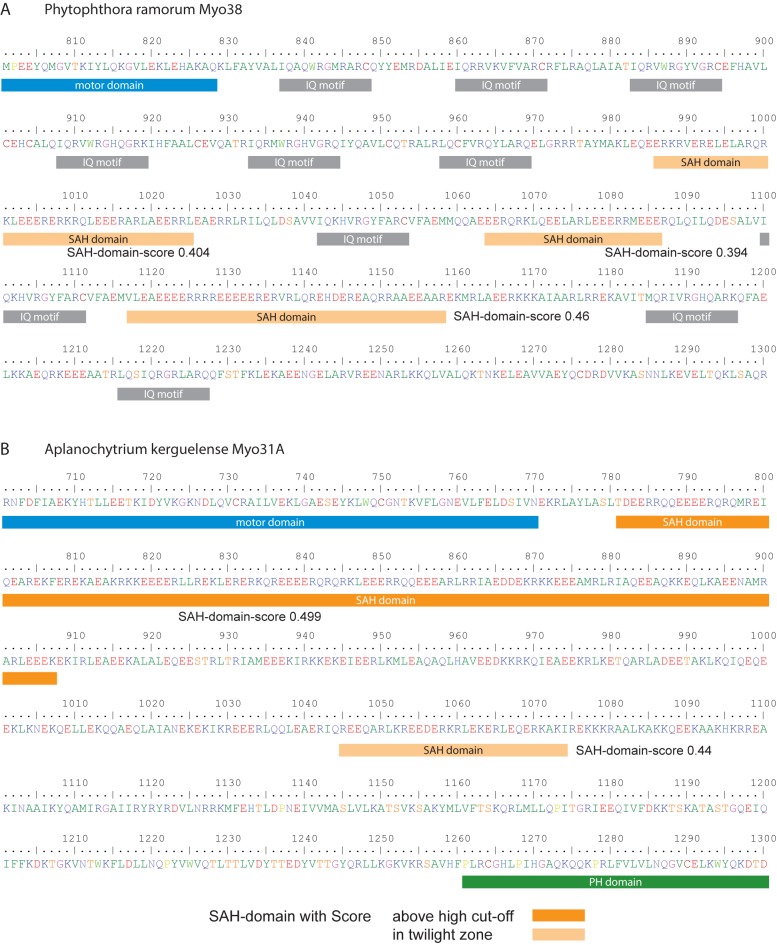
Examples of myosins with unusual lever architectures. A) Tail region of the *Phytophthora ramorum* Myo38 myosin containing alternating IQ-motifs and SAH-domains. For better orientation only the core motifs of the IQ-regions are indicated. For the SAH-domains, the predictions based on the 14 amino acid window were used (S1 Table). B) Tail region of the *Aplanochytrium kerguelense* Myo31A representing the neck region between the motor domain and the C-terminal PH domain. This myosin does not contain any IQ-motif.

In class-38 myosins, we identified alternating IQ-motif regions and SAH-domains (Fig 6A). Multiple SAH-domains have also been determined in many other myosins (Fig 4C, S1 Table) supporting that IQ-motifs and SAH-domains are structural building blocks, which can be used in any order and number to adjust the lever length of myosins. Usually, myosins have at least a single IQ-motif C-terminal to the motor domains acting as canonical lever. Here, we detected a few myosins not containing a single IQ-motif but having SAH-domains directly following the motor domain, for example in the Stramenopiles *Aplanochytrium* Myo31A myosin (Fig 6B) and in an orphan myosin from the cryptophyte *Guillardia theta*.

## Methods

### Determination of SAH-domains in myosins

7919 Myosin sequences were obtained from CyMoBase (www.cymobase.org, [43]), release 19^th^ December 2016, and sorted by class and taxonomy. SAH-domain prediction was performed by querying the Waggawagga API [25]. The prediction provides an SAH-score for each amino acid within a certain window. Here, we used windows with lengths of 14, 21, 28, and 49 amino acids, and computed SAH-scores for each amino acid in each myosin sequence. Although there is no clear distinction between SAH-domains and multimeric α-helical structures (already a point mutation might lead to a switch between these two states), tests with known cases have shown that SAH-regions have scores above 0.25 [25]. We used this rather conservative value to distinguish amino acids within (SAH-score > = 0.25) and outside (SAH-score < 0.25) SAH-regions.

### Determination of SAH-domain-scores

SAH-domains are stretches of continuous amino acids with SAH-scores above 0.25. The criterion for a SAH-domain was to have at least 14 amino acids (around four helical turns). To not obtain regions with multiple predicted SAH-domains interrupted by only one or two residues with SAH-scores below the threshold, we allowed for 20 percent of the SAH-scores of a SAH-domain to be below the threshold. These lower scores were, however, not allowed to be at the start or at the end of the SAH-domain. To provide a score for each SAH-domain but not have peak values dominate the analysis, we defined the SAH-domain-score as the highest average value of the SAH-scores of 14 neighbouring amino acids. The SAH-domain-score depends on the window size used to compute the individual SAH-scores.

For SAH-domain-score based analyses we determined SAH-domain-score cut-offs by sorting the SAH-domain-scores in increasing order, estimating a kernel-regression function to smooth noisy scores, and then determining the cut-off by choosing the SAH-domain-score of the maximum slope change position (the maximum change in the difference between two subsequent SAH-domain-scores).

## Supporting information

S1 Table Lists of predicted SAH-domains. One subtable for each of the four window sizes for computing SAH-scores. In all tables, a SAH length cut-off of 14 amino acids has been applied. One additional subtable for window size of 14 with an SAH length cut-off of ten residues was added for comparison. All subtables contain the name of the myosin sequence, the SAH-domain-score, the SAH-domain sequence, the sequence start and end position of the SAH-domain, the SAH-scores for all residues within the SAH-domains, and the full taxonomy of the species according to NCBI.
(XLS)

S1 Fig Composition of the SAH-score. Helical net view of a SAH-domain region. Some strong and weak interactions are indicated by arrows. The table lists the score of each interaction taken for computing the SAH-score.
(PDF)

S2 Fig Kernel-regression plots to determine SAH-domain-score cut-offs. A separate plot is shown for each window size, with the corresponding SAH-domain-score cut-off.
(PDF)

S3 Fig Distribution of SAH-domains with respect to myosin classes. In contrast to Fig 2, this plot is based on the low SAH-domain-score cut-off for accepting SAH-domains (see S2 Fig). The total number of myosins with SAH-domain is shown for each class. The taxonomic distribution of the myosins with SAH-domains is indicated at the bottom for each class. Note, that the taxons represent the first occurrence of respective myosins with putative SAH-domain, which is not always identical to the first occurrence of the respective myosin class. Subsequently, the SAH-domains were independently lost in many subtaxa so that the respective myosin motor domain and SAH-domain combination is not present in every extant species.
(PDF)

S4 Fig Phylogenetic distribution of the myosins. This figure is similar to Fig 3 but contains the results of all myosin classes. Shortly, all myosins were sorted by taxonomy, and the highest SAH-domain-score for each myosin plotted class by class. The 14 amino acid window size was taken for computing the SAH-score. Major taxa are indicated by colour for better orientation.
(PDF)

S5 Fig Phylogenetic distribution of the myosins. This figure is similar to S4 Fig except that the 49 amino acid window size was taken for computing the SAH-score. Major taxa are indicated by colour for better orientation.
(PDF)

S6 Fig Example for a putative short SAH-domain in class-2 myosins. Alignment of part of the tail region of all human class-2 myosins focusing on the region comprising a predicted short SAH-domain in HsMhc14. For comparison, further mammalian and some other vertebrate Mhc14 homologs are shown indicating that the respective region has evolved after separation of the mammals.
(PDF)
