# Correction: Distribution and evolution of stable single α-helices (SAH domains) in myosin motor proteins

**DOI:** 10.1371/journal.pone.0177716

**Published:** 2017-05-11

**Authors:** Dominic Simm, Klas Hatje, Martin Kollmar

The phrase “Mhc14 class-2 myosins (MYH14, non-muscle myosin 2C)” appears incorrectly throughout the article. The correct phrase should be, “Mhc14 class-2 myosins (MYH7B, myosin cardiac muscle beta chain).” Please see the corrected Fig 5 here.

**Fig 5:**
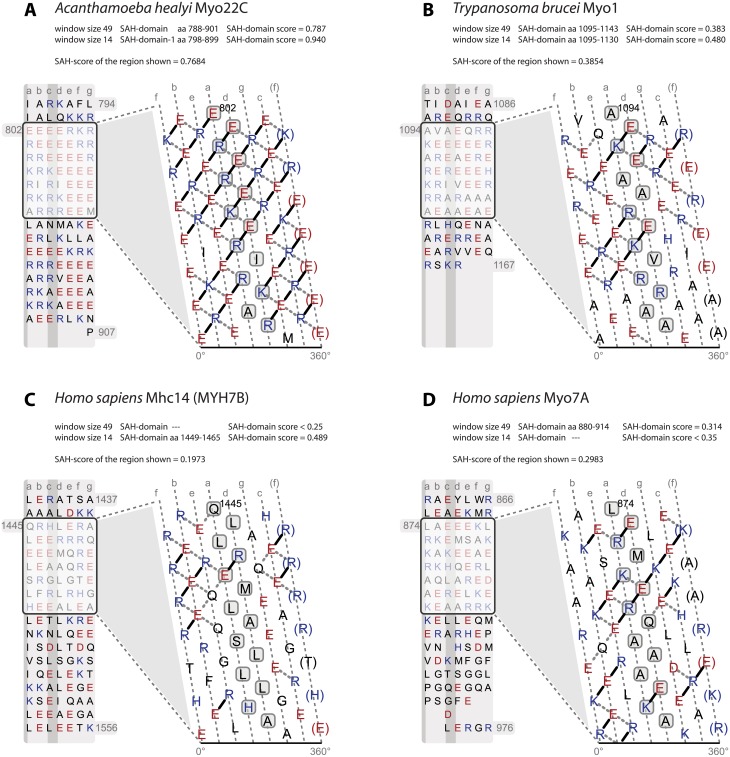
Examples of SAH-domains, and regions probably not containing SAH-domains. A) SAH-domain of *Acanthamoeba healyi* Myo22C, which is among the SAH-domains with the highest SAH-domain-scores. B) Example for a short SAH-domain, which is only detected with the 14, 21, and 28 amino acid window sizes for computing SAH-scores. This short SAH-domain is located C-terminal to the class-1 myosin-specific MyTH1 domain and therefore not part of the lever. Its putative function is to spatially separate the MyTH1 domain from another small domain or protein interaction motif of unknown function at the C-terminus. C) Example for a short SAH-domain with a SAH-domain-score in the twilight zone. This highly charged region is unique to mammalian Mhc14 class-2 myosins (MYH7B, myosin cardiac muscle beta chain; S6 Fig) and interrupts the long coiled-coil filament-forming region of the muscle myosins. The subsequent C-terminal sequence contains a large number of amino acids with high helix-breaking propensity such as glycines and serines. The putative function of the short SAH-domain and the following glycine-rich region might be to open up the coiled-coil. D) Example of a region rich in charged amino acids but with an SAH-domain-score in the range of non-SAH-domains.
