# Supplementary material for: Distribution and evolution of stable single α-helices (SAH domains) in myosin motor proteins
Source: PLoS One. 2017 Apr 3;12(4):e0174639. doi: 10.1371/journal.pone.0174639 (PMC5378345; doi:10.1371/journal.pone.0174639)
Supplement: S1 Fig — Helical net view of a SAH-domain region. Some strong and weak interactions are indicated by arrows. The table lists the score of each interaction taken for computing the SAH-score. (PDF) [file pone.0174639.s002.pdf]

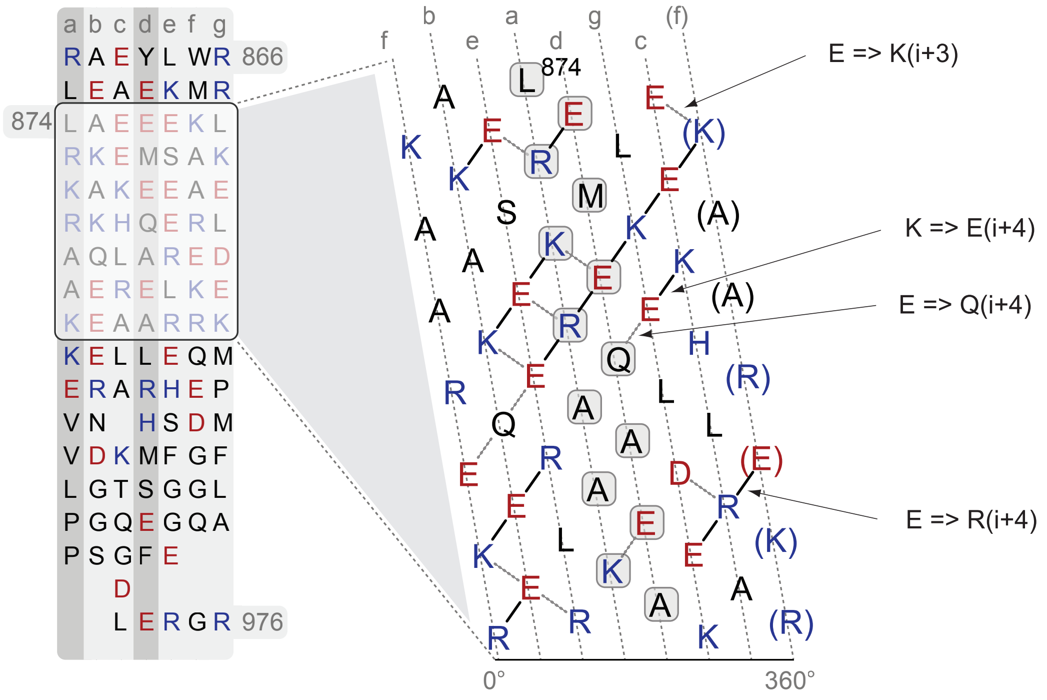

Stabilization / destabilization by amino acid interactions

Source residue	Target residue	Interaction	Score	Stabilization
H/K/R	E	i+4	1.0	strong
E	K/R	i+4	1.0	strong
H/K/R	E	i+3	0.75	medium
E	K/R	i+3	0.75	medium
K/R	D	i+4	0.75	medium
K/R	D	i+3	0.5	weak
D	K/R	i+4	0.75	medium
D	K/R	i+3	0.5	weak
E	H	i+3	0.5	weak
Q	E	i+4; i+3	0.5	weak
E	Q	i+4; i+3	0.5	weak
network stabilization		i, i+3, i+6	1.5	network
(oppositely charged residues)		i, i+3, i+7	0.25	network
		i, i+4, i+7	0.25	network
		i, i+4, i+8	2.0	network
hydrophobic seam		i, i+3, i+6	-0.5	destabilizing
		i, i+3, i+7	-0.5	destabilizing
		i, i+4, i+7	-0.5	destabilizing
		i, i+4, i+8	-0.5	destabilizing

Stabilization / destabilization by helix-forming propensity

Residue	Score	Stabilization
A	0.25	stabilizing
G	-0.5	destabilizing
P	-0.75	destabilizing
