# Supplementary figures and images for: Distribution and evolution of stable single α-helices (SAH domains) in myosin motor proteins
Source: PLoS One. 2017 Apr 3;12(4):e0174639. doi: 10.1371/journal.pone.0174639 (PMC5378345; doi:10.1371/journal.pone.0174639)

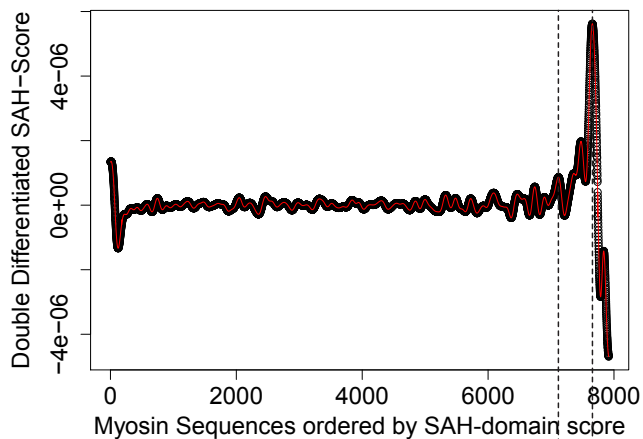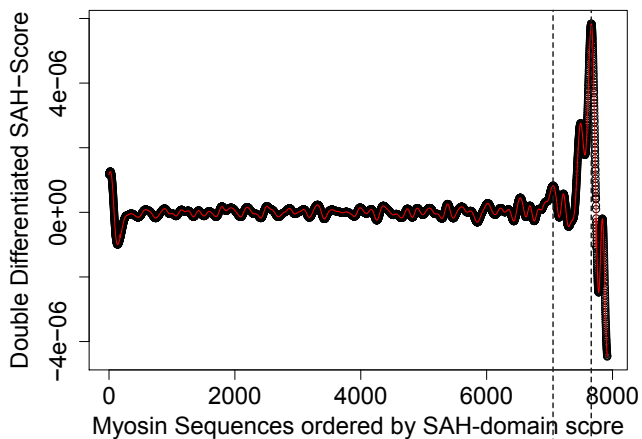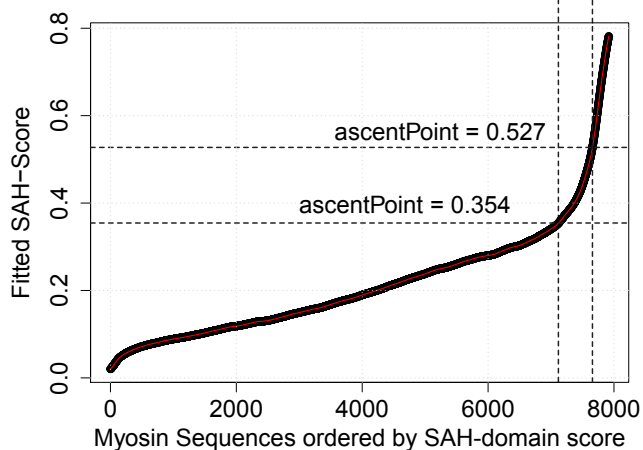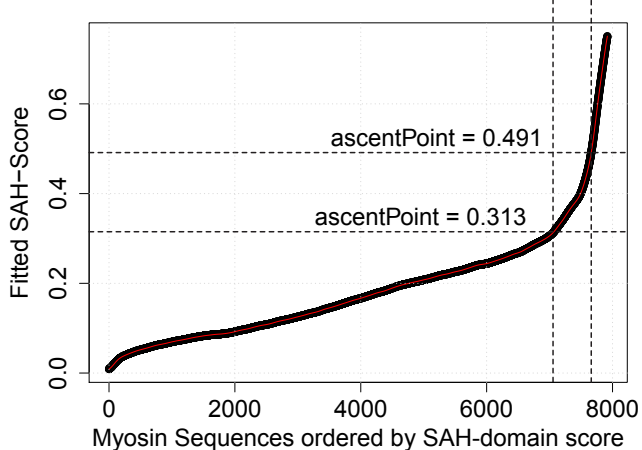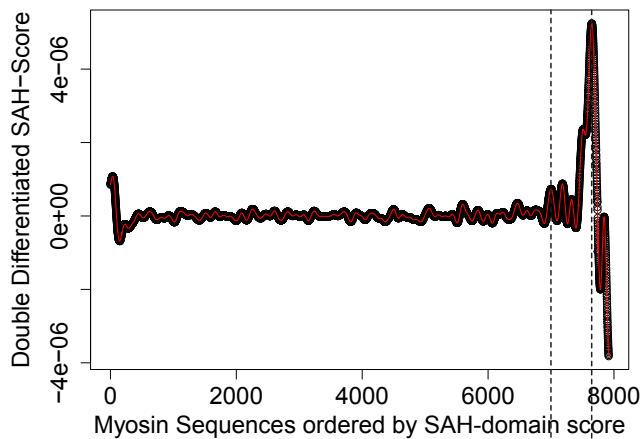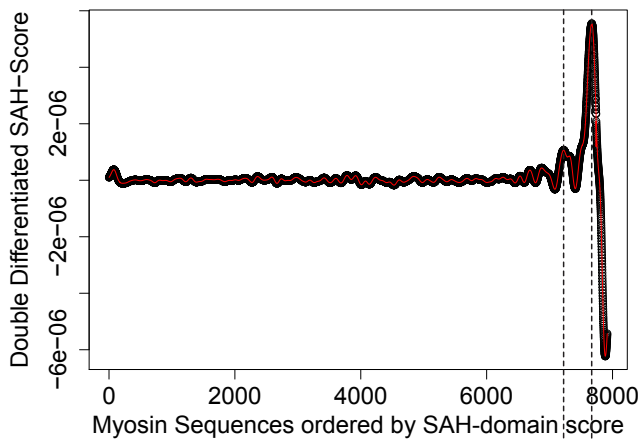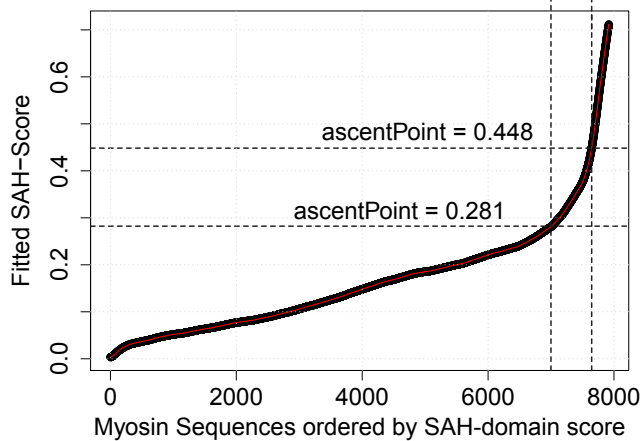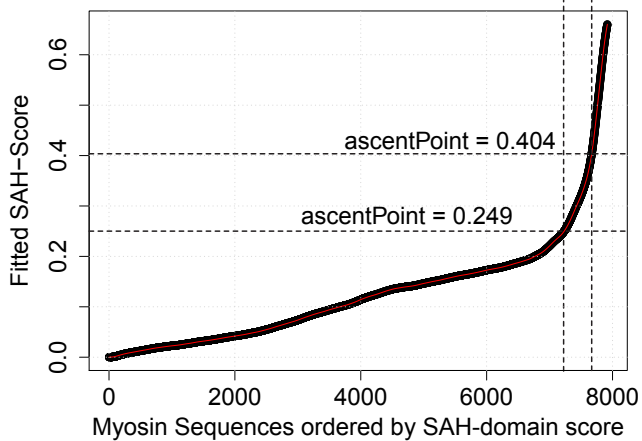

Supplement: S2 Fig — A separate plot is shown for each window size, with the corresponding SAH-domain-score cut-off. (PDF) [file pone.0174639.s003.pdf]

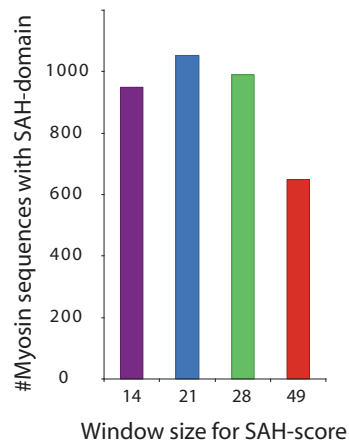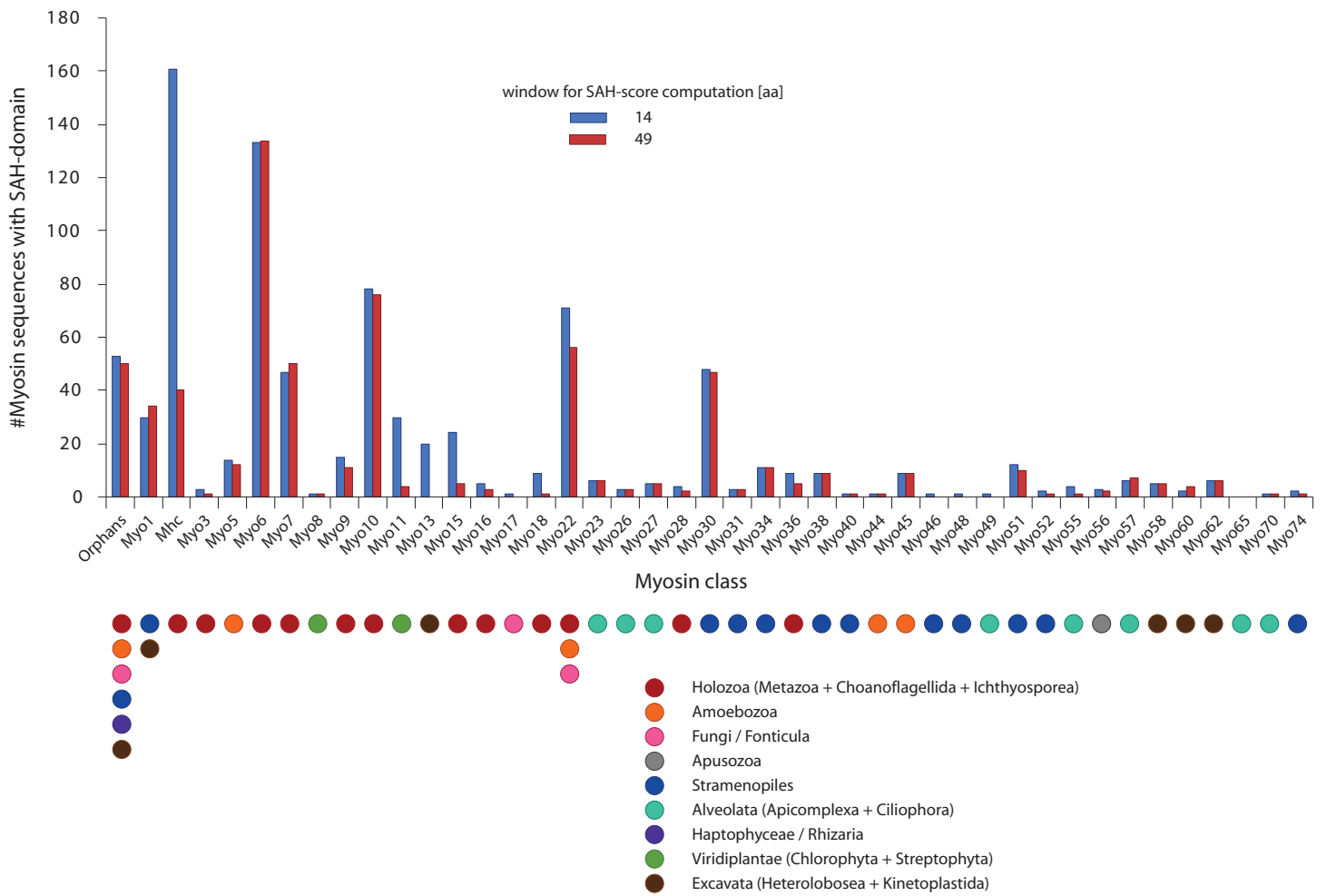

Supplement: S3 Fig — In contrast to Fig 2, this plot is based on the low SAH-domain-score cut-off for accepting SAH-domains (see S2 Fig). The total number of myosins with SAH-domain is shown for each class. The taxonomic distribution of the myosins with SAH-domains is indicated at the bottom for each class. Note, that the taxons represent the first occurrence of respective myosins with putative SAH-domain, which is not always identical to the first occurrence of the respective myosin class. Subsequently, the SAH-domains were independently lost in many subtaxa so that the respective myosin motor domain and SAH-domain combination is not present in every extant species. (PDF) [file pone.0174639.s004.pdf]

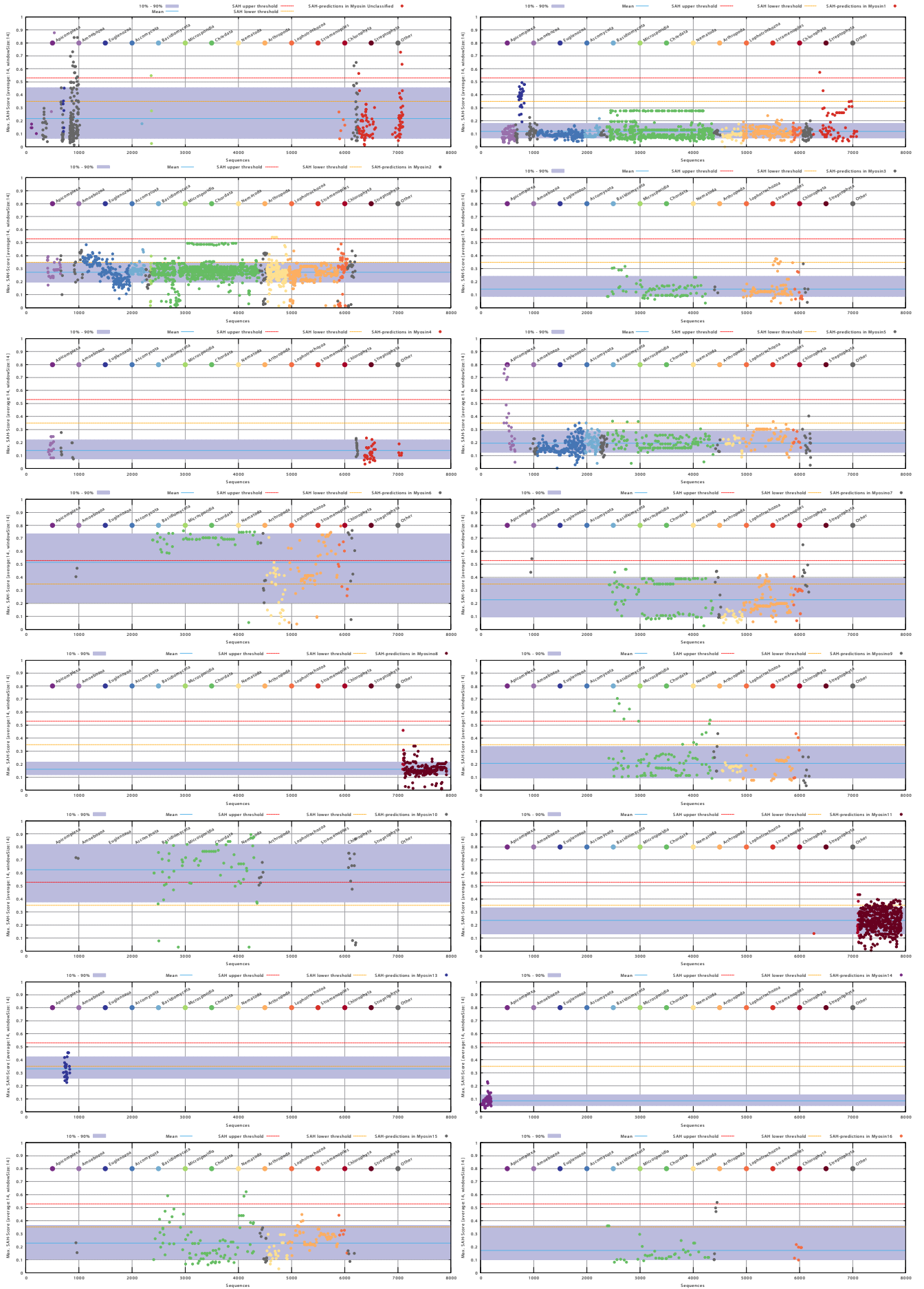



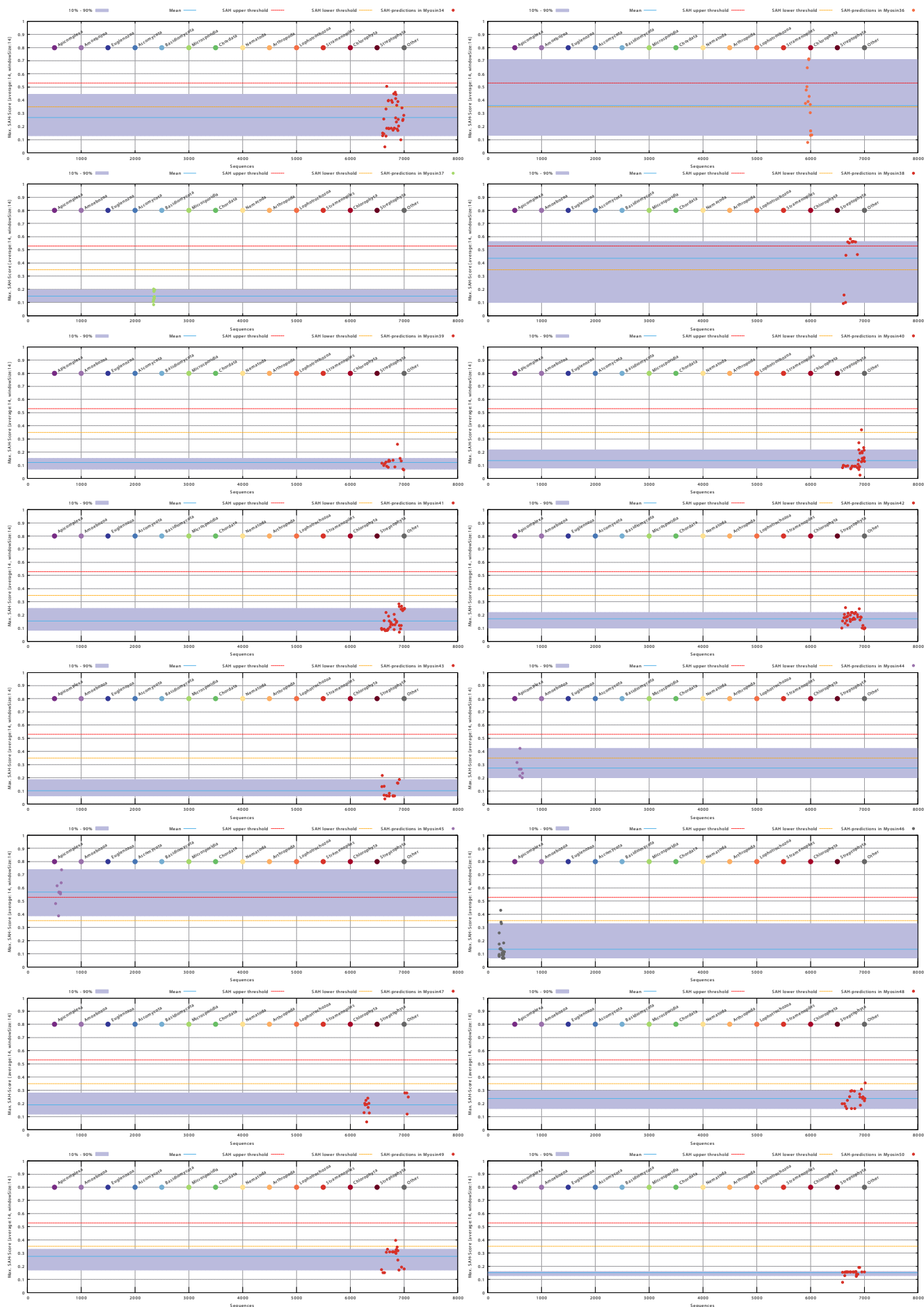

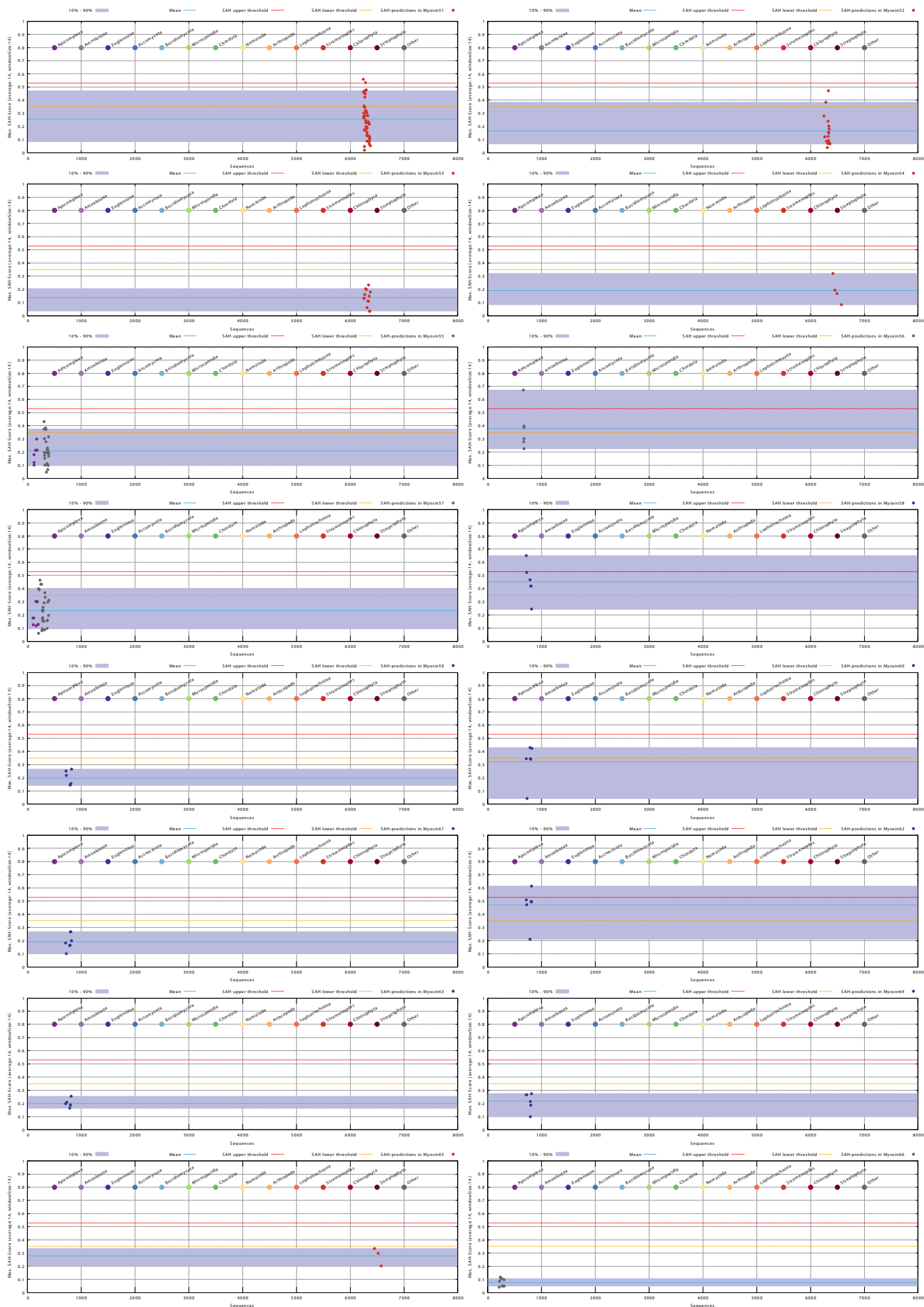

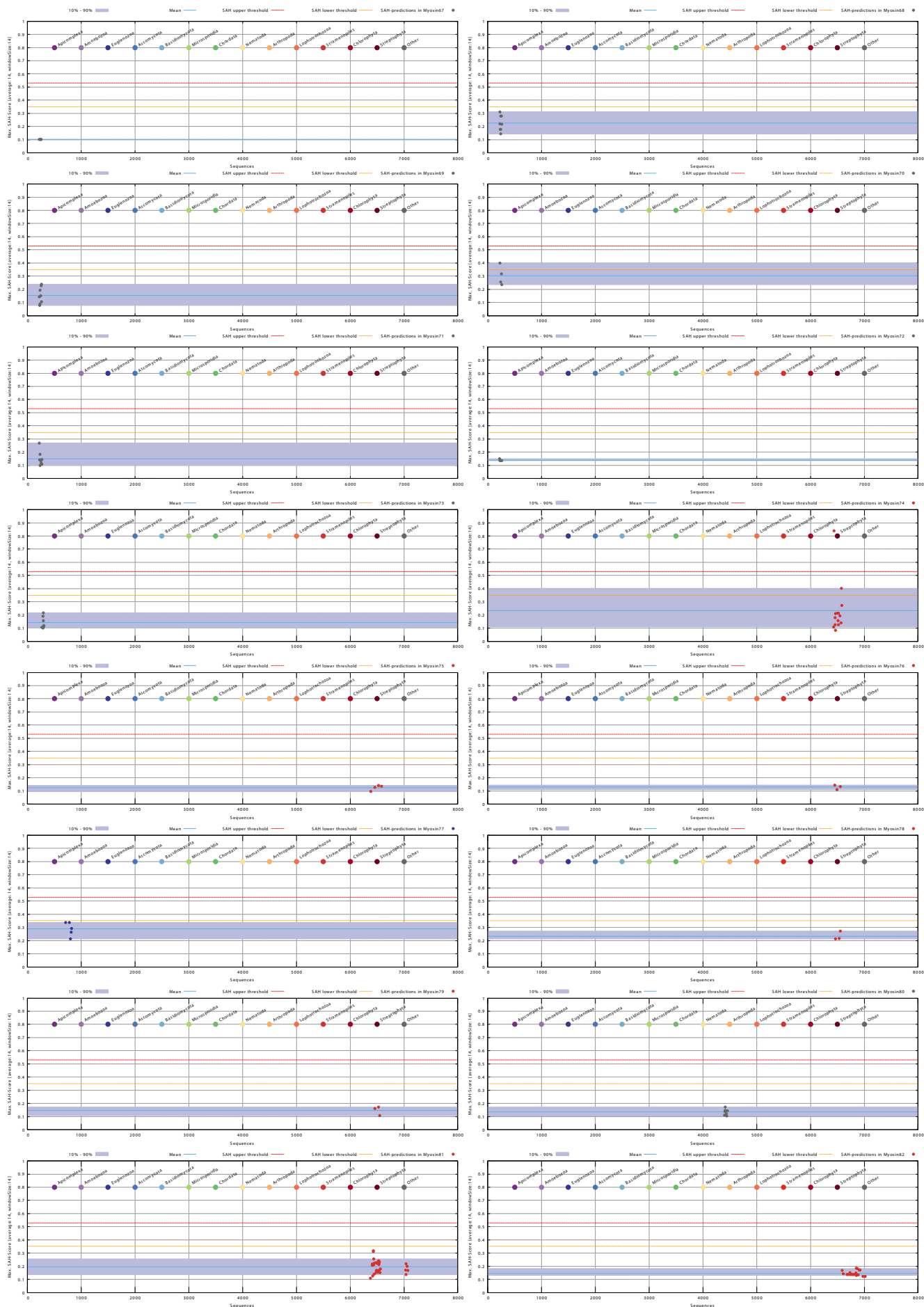

Supplement: S4 Fig — This figure is similar to Fig 3 but contains the results of all myosin classes. Shortly, all myosins were sorted by taxonomy, and the highest SAH-domain-score for each myosin plotted class by class. The 14 amino acid window size was taken for computing the SAH-score. Major taxa are indicated by colour for better orientation. (PDF) [file pone.0174639.s005.pdf]

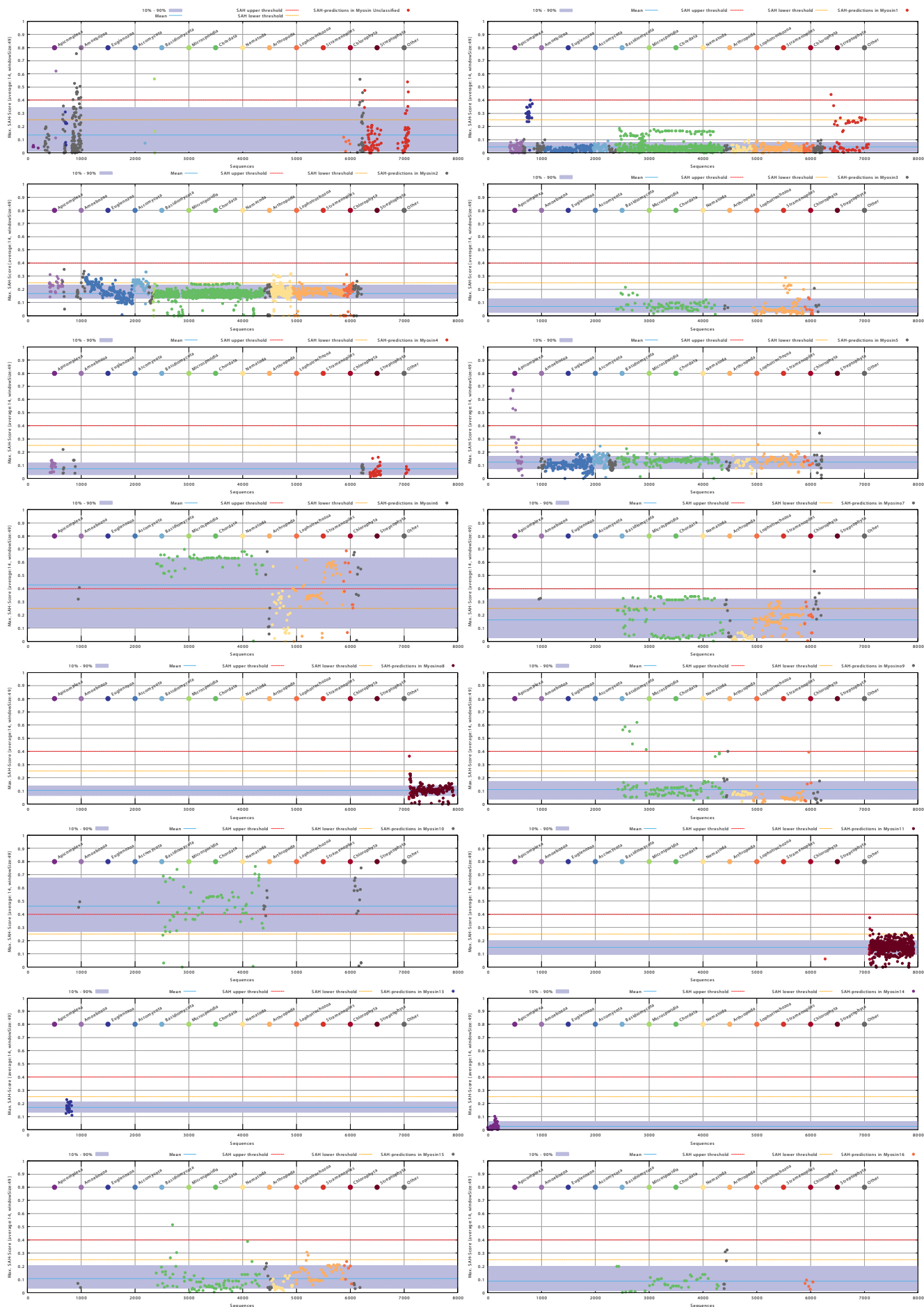

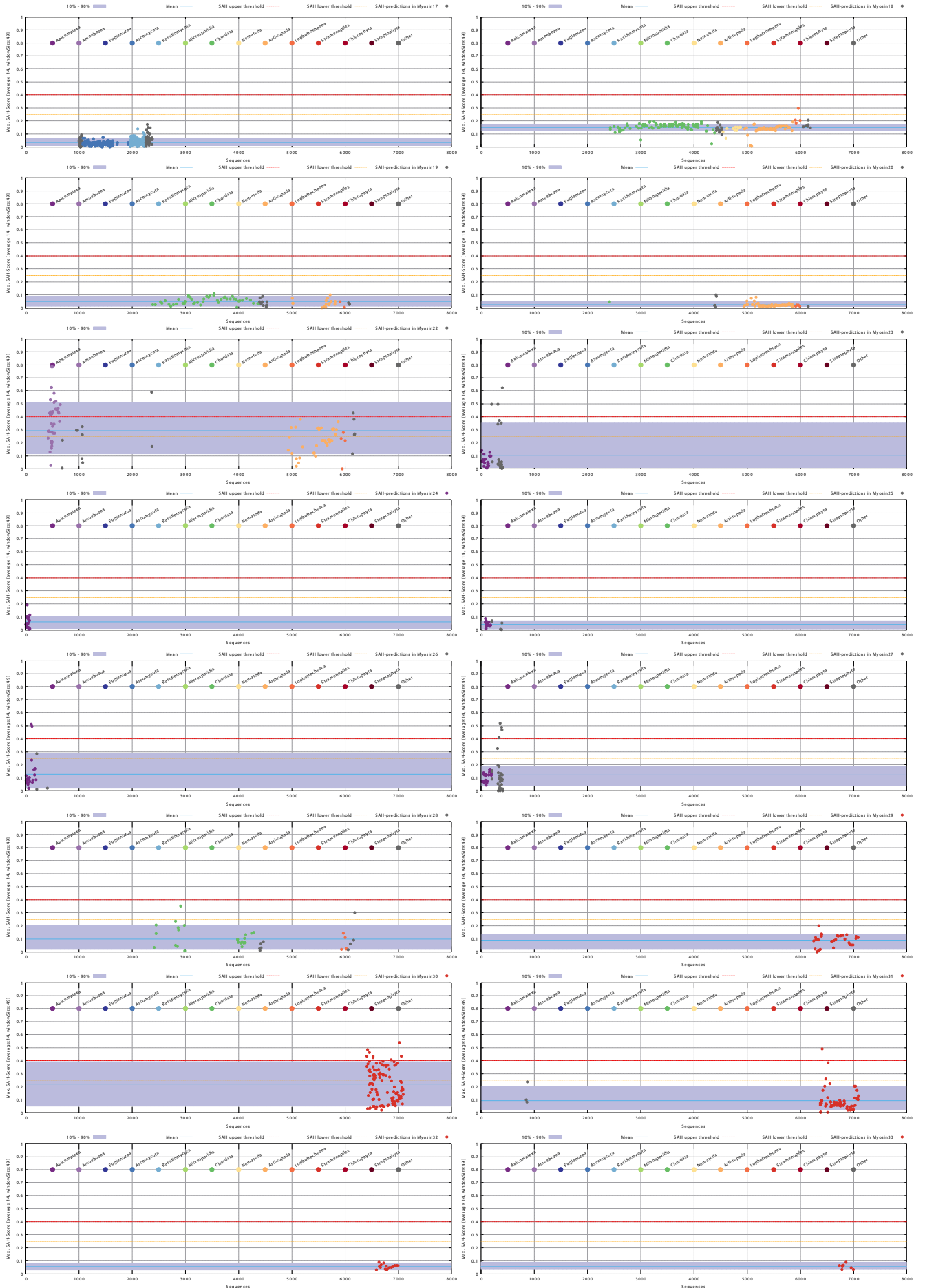

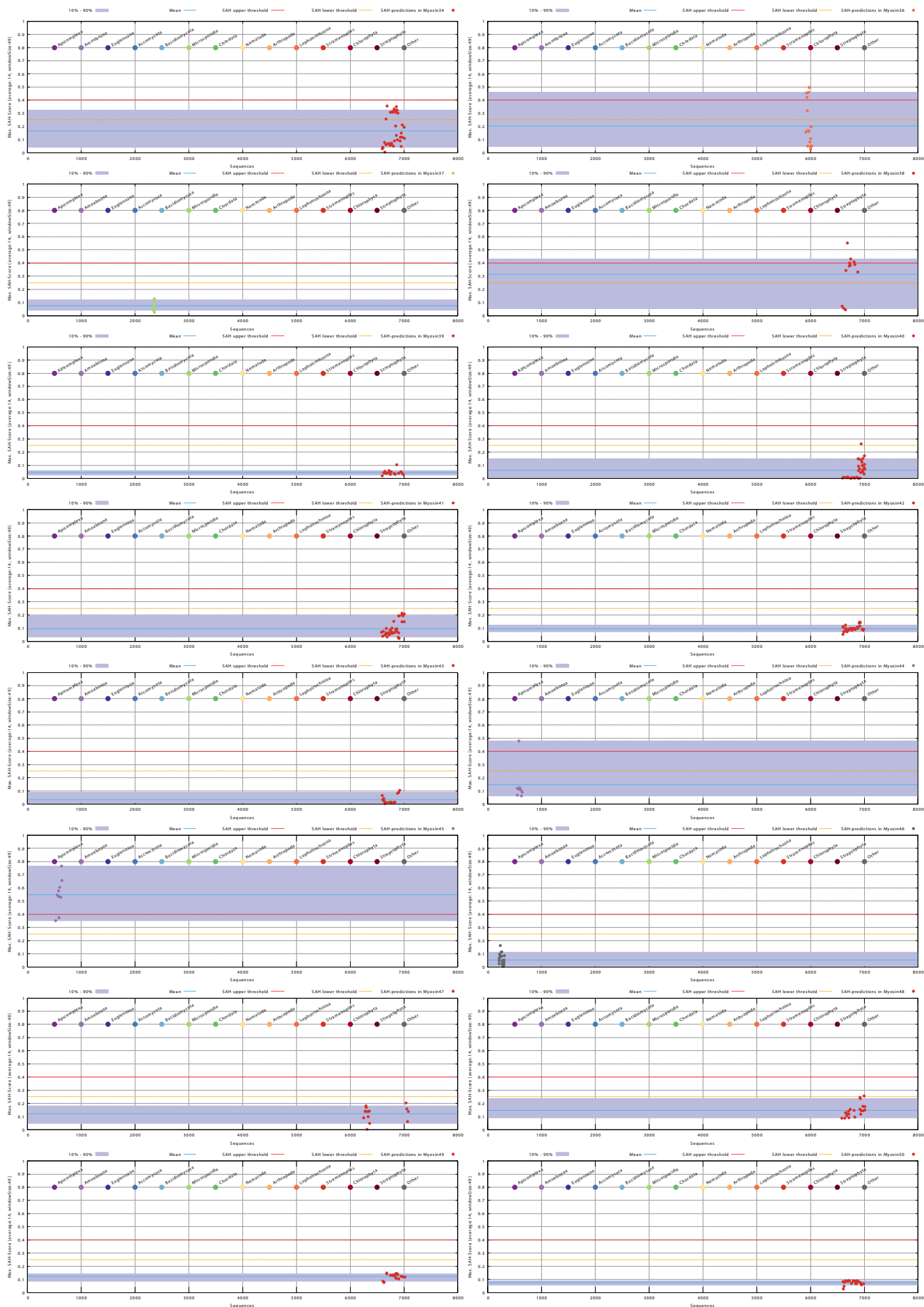



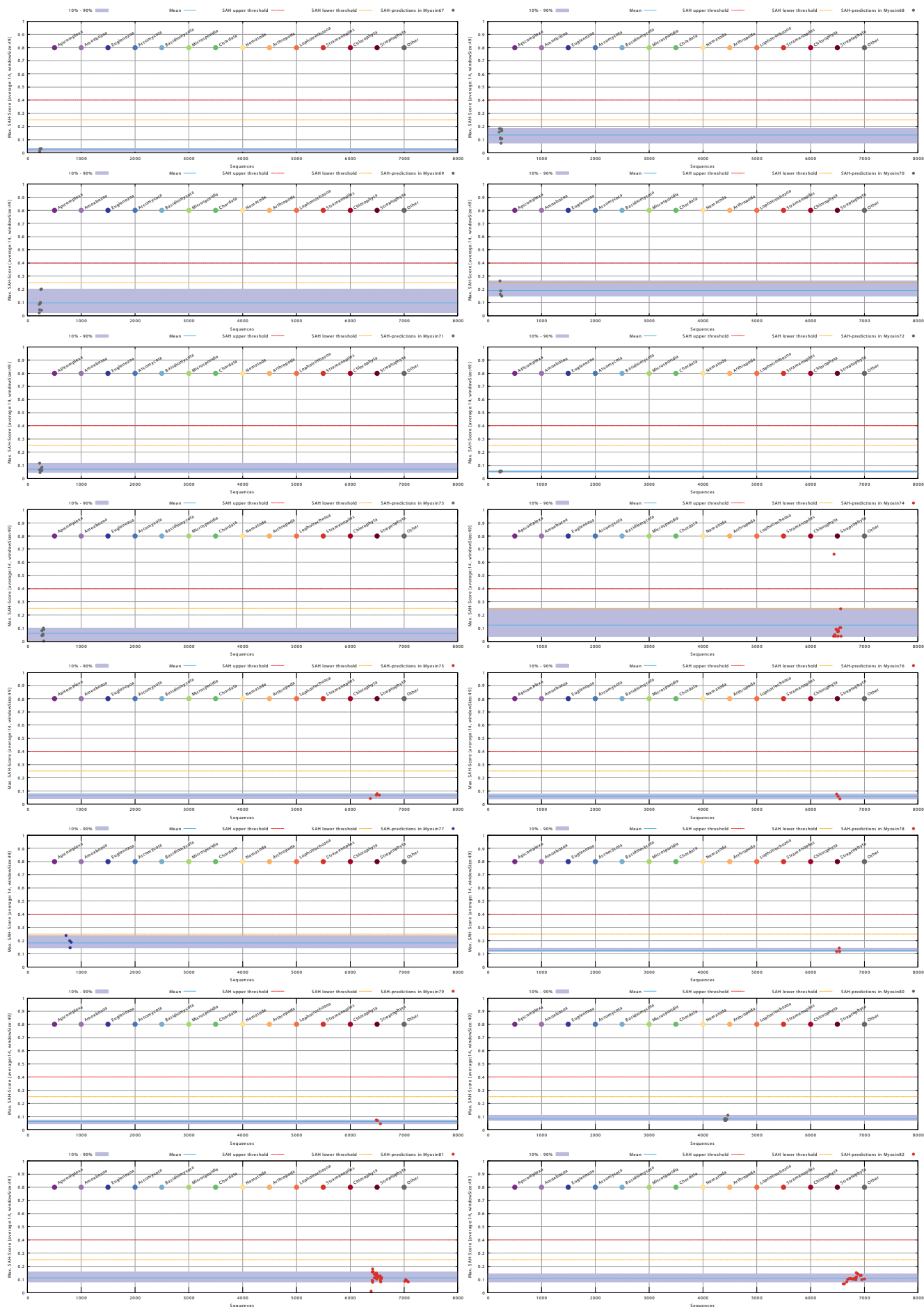

Supplement: S5 Fig — This figure is similar to S4 Fig except that the 49 amino acid window size was taken for computing the SAH-score. Major taxa are indicated by colour for better orientation. (PDF) [file pone.0174639.s006.pdf]
