# Supplementary material for: Distribution and evolution of stable single α-helices (SAH domains) in myosin motor proteins
Source: PLoS One. 2017 Apr 3;12(4):e0174639. doi: 10.1371/journal.pone.0174639 (PMC5378345; doi:10.1371/journal.pone.0174639)
Supplement: S6 Fig — Alignment of part of the tail region of all human class-2 myosins focusing on the region comprising a predicted short SAH-domain in HsMhc14. For comparison, further mammalian and some other vertebrate Mhc14 homologs are shown indicating that the respective region has evolved after separation of the mammals. (PDF) [file pone.0174639.s007.pdf]

```
HsMhc14 numbering

                1420      1430      1440      1450      1460      1470      1480
                |....|....|....|....|....|....|....|....|....|....|....|....|....|....
HsMhc1     KTKQRLQNEVEDLMIDVERTNAACAALDKKQRNFDKILAEWKQKCEETHAELEASQKESRSLSTELFKIK
HsMhc2     KTKQRLQNEVEDLMLDVERTNAACAALDKKQRNFDKILAEWKQKCEETHAELEASQKEARSLGTELFKIK
HsMhc4     KTKQRLQNEVEDLMIDVERSNAACIALDKKQRNFDKVLAEWKQKYEETQAELEASQKESRSLSTELFKVK
HsMhc8     KTKQRLQNEVEDLMLDVERSNAACAALDKKQRNFDKVLSEWKQKYEETQAELEASQKESRSLSTELFKVK
HsMhc3     KTKQRLQGEVEDLMVDVERANSLAAALDKKQRNFDKVLAEWKTKCEESQAELEASLKESRSLSTELFKLK
HsMhc13    KTKQRLQGEVEDLMRDLERSHTACATLDKKQRNFDKVLAEWKQKLDESQAELEAAQKESRSLSTELFKMR
HsMhc6     KTKHRLQNEIEDLMVDVERSNAAAAALDKKQRNFDKILAEWKQKYEESQSELESSQKEARSLSTELFKLK
HsMhc7     KTKHRLQNEIEDLMVDVERSNAAAAALDKKQRNFDKILAEWKQKYEESQSELESSQKEARSLSTELFKLK
HsMhc14    KAKLRLQTESEDVTLELERATSAAAALDKKQRHLERALEERRRQEEEMQRELEAAQRESRGLGTELFRLR
MmMhc14    KAKLRLQTESEDVTLELERATSAAAALDKKQRHLERALEERRRQEEEMQRELEAAQREARGLGTELFRLR
BtMhc14    KAKLRLQTESEDVTLELERATSAAAALDKKQRHLERALEERRRQEEEMQRELEAAQREARSLGTELFRLR
GgMhc14    KTKHRLQTEIEDLSVDLERANSACAALDKKQRNFDRILAEWKQKYEETQAELEASQKESRSLSTELFKLK
XtMhc14    KTKHRLQTEIEDLVIDLERANSAAAALDKKQRNFDRILGEWKQKYEETQAELEASQKESRSLSTELFKLK
BrMhc14A   KTKHRLQTEIEDLMVDLERSNAVAIALDKKQRNFDKVLSEWRQKFEETQSELEGSQKESRSLSTELFKLK
HsMhc15    RARHQLQLELGDALSDLGKVRSAAARLDQKQLQSGKALADWKQKHEESQALLDASQKEVQALSTELLKLK
HsMhc20    KNKQRLQAEVEDLTIDLEKANAAAAALDKKQRLFDKMLAEWQQKCEELQVEVDSSQKECRMYMTESFKIK
HsMhc16    RGRRRLQQELDDATMDLEQQRQLVSTLEKKQRKFDQLLAEEKAAVLRAVEERERAEAEGREREARALSLT
HsMhc10    KTKNRLQQELDDLTVDLDHQRQVASNLEKKQKKFDQLLAEEKSISARYAEERDRAEAEAREKETKALSLA
HsMhc11    KTKNRLQQELDDLVVDLDNQRQLVSNLEKKQRKFDQLLAEEKNISSKYADERDRAEAEAREKETKALSLA
HsMhc9     KTKTRLQQELDDLLVDLDHQRQSACNLEKKQKKFDQLLAEEKTISAKYAEERDRAEAEAREKETKALSLA
           ..a..d...a..d...a..d...a..d...a..d...a..d...a..d...a..d...a..d...a..d.
```

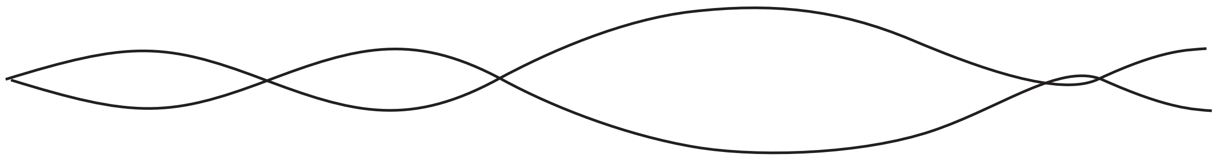

- Hs *Homo sapiens*
- Mm *Mus musculus*
- Bt *Bos taurus*
- Gg *Gallus gallus*
- Xt *Xenopus tropicalis*
- Br *Brachydano rerio*
